# Molecular cytogenetic analysis of genome-specific repetitive elements in *Citrus clementina* Hort. Ex Tan. and its taxonomic implications

**DOI:** 10.1186/s12870-019-1676-3

**Published:** 2019-02-15

**Authors:** Honghong Deng, Suqiong Xiang, Qigao Guo, Weiwei Jin, Zexi Cai, Guolu Liang

**Affiliations:** 1 College of Horticulture and Landscape Architecture, Southwest University, Chongqing, 400715 China; 2 National Maize Improvement Center, College of Agronomy and Biotechnology, China Agricultural University, Beijing, 100193 China

**Keywords:** Repetitive DNA sequences, Satellite DNA, rDNA, Multicolor FISH, Karyotype, Hybrid identification

## Abstract

**Background:**

Clementine mandarin (*Citrus clementina* Hort. ex Tan.) is one of the most famous and widely grown citrus cultivars worldwide. Variations in relation to the composition and distribution of repetitive DNA sequences that dominate greatly in eukaryote genomes are considered to be species-, genome-, or even chromosome-specific. Repetitive DNA-based fluorescence in situ hybridization (FISH) is a powerful tool for molecular cytogenetic study. However, to date few studies have involved in the repetitive elements and cytogenetic karyotype of Clementine.

**Results:**

A graph-based similarity sequence read clustering methodology was performed to analyze the repetitive DNA families in the Clementine genome. The bioinformatics analysis showed that repetitive DNAs constitute 41.95% of the Clementine genome, and the majority of repetitive elements are retrotransposons and satellite DNAs. Sequential multicolor FISH using a probe mix that contained CL17, four satellite DNAs, two rDNAs and an oligonucleotide of (TTTAGGG)_3_ was performed with Clementine somatic metaphase chromosomes. An integrated karyotype of Clementine was established based on unequivocal and reproducible chromosome discriminations. The distribution patterns of these probes in several *Citrus*, *Poncirus* and *Fortunella* species were summarized through extensive FISH analyses. Polymorphism and heterozygosity were commonly observed in the three genera. Some asymmetrical FISH loci in Clementine were in agreement with its hybrid origin.

**Conclusions:**

The composition and abundance of repetitive elements in the Clementine genome were reanalyzed. Multicolor FISH-based karyotyping provided direct visual proof of the heterozygous nature of Clementine chromosomes with conspicuous asymmetrical FISH hybridization signals. We detected some similar and variable distribution patterns of repetitive DNAs in *Citrus*, *Poncirus*, and *Fortunella*, which revealed notable conservation among these genera, as well as obvious polymorphism and heterozygosity, indicating the potential utility of these repetitive element markers for the study of taxonomic, phylogenetic and evolutionary relationships in the future.

**Electronic supplementary material:**

The online version of this article (10.1186/s12870-019-1676-3) contains supplementary material, which is available to authorized users.

## Background

Clementine mandarin (*Citrus clementina* Hort. ex Tan.) (2n = 2x = 18) [1], belonging to the Rutaceae family [2], is one of the most famous commercial citrus cultivars worldwide [3, 4]. Because of its vital importance in human diets, commercial profits and breeding programs, Clementine has received increasing attention of consumers, growers and breeders throughout the world [3–5], particularly in some Mediterranean countries, such as Spain, Italy and France, as well as in Argentina, Uruguay, South Africa and Peru [4, 6].

Repetitive DNA sequences represent a large proportion of the genome in higher eukaryotes [7], reaching 20% or, in some cases, up to 90% of the genome size [8], and they comprise tandem repeat sequences (satellites, minisatellites and microsatellites) and dispersed transposable elements (transposons and retrotransposons) [9]. Repetitive DNAs can be species- or genome-specific or even chromosome-specific in many species within a taxonomic family or diverse taxa, as some repeats are highly-conserved while others are the evolutionarily fastest parts of the genome, showing pronounced differences even between closely related species [8–10]. For a long time, repetitive DNAs were assumed to be ‘junk’ or, even worse, ‘selfish’ DNA [11]. Many recent studies have emphasized the important role of repetitive DNAs in determining the size, composition and evolution of genomes. Therefore, knowledge of the molecular characterization of the structure and organization of repetitive DNAs is essential. From a computational perspective, repeats have always presented technical challenges for sequence alignment and genome assembly programs [12].

Karyotypes can yield valuable information regarding the highest level of structural and functional organization of chromosomes that are the main carriers of genetic material within the nuclei of each eukaryotic cell [13]. The more closely related species are, the more similar karyotypes they share [14]. Karyotype features are irrelevant to internal gene expression, external environmental conditions and other confounding factors [15], which can represent an isolating mechanism and a major driver in the process of speciation and macroevolution [13, 16]. Taken together, karyotype can provide an excellent opportunity for taxonomic and phylogenetic analysis [13–16]. Recently, the mapping of repetitive DNAs by fluorescence in situ hybridization (FISH) has been successfully applied to many karyotype studies, especially when addressing related species [17–20].

With the increasing availability of simple sequence repeat (SSR) markers, expressed sequence tag (ESTs) databases, gene microarray comparative analysis, and physical and genetic mapping studies, the International Citrus Genome Consortium (ICGC) has successfully released the genome sequence of the Clementine as a primary reference genome for *Citrus* and related genera into the future [4]. However, the repetitive elements and cytogenetic karyotype of Clementine are poorly characterized.

The highly repetitive DNA sequences of a few citrus varieties have been successfully characterized by CsCl-actinomycin D gradients or molecular cloning during the last decade, including *C. ichangensis* Swing. [21], *C. limon* (L.) Burm [22, 23], *C. sinensis* [21, 24], *Poncirus trifoliata* [21, 22], and *C. sunki* [25]. Nonetheless, their specific physical hybridization locations in chromosomes are still not well defined [26]. Wu et al. sequenced and compared the genomes of several citrus varieties, but did not show satellite DNAs in their article [3].

The primary aim of the present study was to develop an efficient and precise approach for the identification of individual chromosomes in Clementine based on genome-specific repetitive DNA sequences and construct a standard integrated karyotype. Hence, we performed bioinformatics analysis to characterize the repetitive elements in the Clementine genome. The cytological locations of major tandemly repeated DNAs were revealed by sequential multicolor FISH along with a few other repeats as probe cocktails, resulting in a fine-tuned Clementine karyotype. Additionally, we explored the potential applicability of these probes for hybrid identification and the study of taxonomical relationships within some species belonging to *Citrus*, *Poncirus*, and *Fortunella*. Finally, our research results will add to the global knowledge base related to *Citrus* genetics and breeding.

## Results

### Repetitive DNA composition and abundance in *C. clementina* and identification of satellite repeats

A total of 379 clusters (Additional file 1) were produced with a cluster size threshold of 0.01%. The bioinformatics analysis data revealed that the Clementine genome, like other higher eukaryotic plant genomes, contained a large proportion of different families of repetitive DNA elements. The observed proportions of each family of repetitive DNAs in the Clementine genome are summarized in Table 1, and the specific information is listed in Additional file 1.

**Table 1:** Different families of repetitive DNAs and their proportions in the *C. clementina* Hort. ex Tan. genome

Repeat element	proportion %
Retrotransposon	**22.74**
LTR.Gypsy	12.60
LTR.Copia	7.91
LTR.Caulimovirus	1.45
Unclassified LTR	0.08
LINE.L1	0.49
LINE.Penelope	0.18
LINE.RT	0.03
DNA Transposon	**1.31**
DNA.CMC.EnSpm	0.44
DNA.hAT.Ac	0.12
DNA.hAT.Tip100	0.01
DNA.MULE.MuDR	0.48
DNA.PIF.Harbinger	0.01
DNA.TcMar.Stowaway	0.23
RC.Helitron	0.02
rDNA	**2.93**
Satellite	**9.28**
Simple_repeat	**1.80**
Low_complexity repeats	**1.87**
Unclassified repeats	**2.08**
Total	**41.95**

Bold number represents the total proportion of each type of repeat elements

The total quantitative amount of repetitive DNAs accounted for 41.95% of the whole Clementine genome, including retrotransposons (LTR.Gypsy, LTR.Copia, LTR.Caulimovirus, Uclassified LTR, LINE.L1, and LINE.Penelope,LINE.RT), transposons (DNA.CMC.EnSpm, DNA.hAT.Ac, DNA.hAT.Tip100, DNA.MULE.MuDR, DNA.PIF.Harbinger, DNA.TcMar.Stowaway, and RC.Helitron), rDNA, satellites DNAs, simple repeats, low-complexity repeats and some unclassified repeats. The majority of the repetitive elements in Clementine are retrotransposons and satellite DNAs, constituting 22.40 and 11.01% of the whole genome, respectively. Among LTR-retrotransposons, Ty3/Gypsy elements were the predominant repetitive elements, making up 12.60% of the total, and exceeding Ty1/copia by approximately 1.59-fold in terms of their proportions in the genome. Other LTR-retrotransposons were found to compose relatively smaller genome proportions, including LTR.Caulimovirus with 1.45%, LINE with 0.70% and unclassified LTR with 0.08%. DNA transposons accounted for only 1.31% of the Clementine genome. The DNA.MULE.MuDR content was 0.475%, and it was the dominant component among DNA transposons. The rDNA elements occupied 2.93% of the Clementine genome. Simple repeats and low-complexity repeats represented 1.80 and 1.87%, respectively. The estimation of repetitive DNA abundance showed that 2.08% of the repetitive elements were unclassified (Table 1).

Four major satellite DNAs in Clementine were identified via bioinformatics analysis, CL1, CL2, CL3 and CL4, which accounted for 3.16, 3.01, 1.55 and 1.21% of the genome, respectively (Additional file 2). We isolated and cloned the four major satellite DNAs, two rDNAs and a centromere-specific retrotransposon sequence, CL17, from the Clementine genome. The specific primers for amplifying these repeats are shown in Additional file 3.

### Chromosomal mapping of repetitive DNAs in *C. clementina*

A probe mix that contained CL17, four major satellite DNAs, two rDNAs and a telomeric repeat in Clementine was hybridized onto its own somatic metaphase chromosomes prepared from root tips. To obtain more detailed and accurate information, FISH hybridization signals were obtained from these probes in three independent trials, each comprising five slides of chromosome preparations. At least 15 spreads of mitotic metaphase chromosomes were analyzed on each slide.

**Fig. 1:**
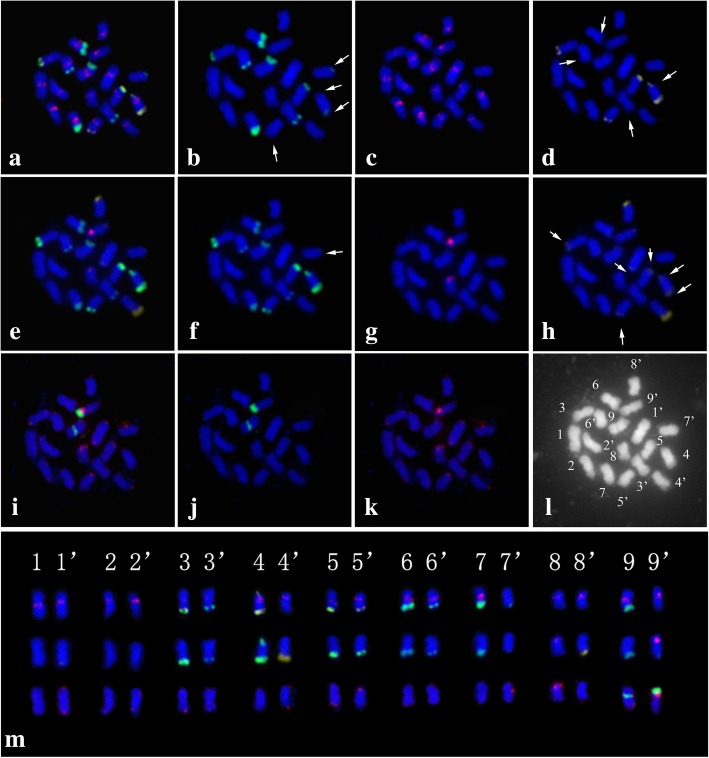
Multicolor FISH identification using a probe mix of *C. clementina* somatic metaphase chromosomes that were successively hybridized onto the same metaphase plate. The chromosomes were counterstained with DAPI (blue), and probe signals were pseudocolored in different colors (red, green and yellow). For a better visualization, the FISH signals in (**b**–**d**), (**f**–**h**), and (**j**–**k**) were digitally separated from the merged graphs (**a**), (**e**) and (**i**), respectively. (**b**) CL1; (**c**) CL17; (**d**) CL3; (**f**) CL2; (**g**) 5 s rDNA; (**h**) CL4; (**j**) 45 s rDNA; (**k**) TTTAGGG; (**l**) image showing the morphology of metaphase chromosomes; (**m**) the chromosomes in the karyogram extracted from the respective photomicrographs (**a**), (**e**), and (**i**), were numbered and ordered on the basis of their total descending length, FISH signals and morphological characteristics. Individual chromosome pairs of *C. clementina* were discriminated after three rounds of multicolor FISH

The chromosome number was determined and matched the expected chromosome number for Clementine of 2n = 2x = 18. CL17 generated obvious FISH hybridization signals in the centromeric or pericentromeric regions on each chromosome of Clementine, and no additional signal was detected anywhere else (Fig. 1c). Telomeric repeats produced clear hybridization signals at the terminal positions of each chromosome (Fig. 1k).

The signals of the four satellite DNAs exhibited diverse distribution patterns regarding the numbers, positions and intensities of hybridization signals on the chromosomes. The differential brightness of FISH hybridization signals on individual somatic chromosomes represented the distinct intensities of signals (Fig. 1b, d, f, and h). The CL1 and CL2 probes showed 10 similar signals adjacent to the subterminal and terminal positions of the long and short arms of the chromosomes and one unique signal on each homolog of chromosome pair 9, respectively (Fig. 1b, f). The CL3 and CL4 probes generated 8 and 7 signals, respectively (Fig. 1h, d). Although the four different tandem repeats had different distribution patterns, simultaneous hybridization analysis revealed that they were co-localized on one or more pairs of chromosomes with partially or completely overlapping signals in Clementine (Fig. 1a, e). Clementine also exhibited a highly heterozygous rDNA locus bearing a pair of co-localized 5 s–45 s-rDNA sites, a single 5 s and a single 45 s rDNA site (Fig. 1g, j).

### Karyotyping analysis of *C. clementina* based on multicolor FISH with mitotic metaphase chromosomes

**Fig. 2:**
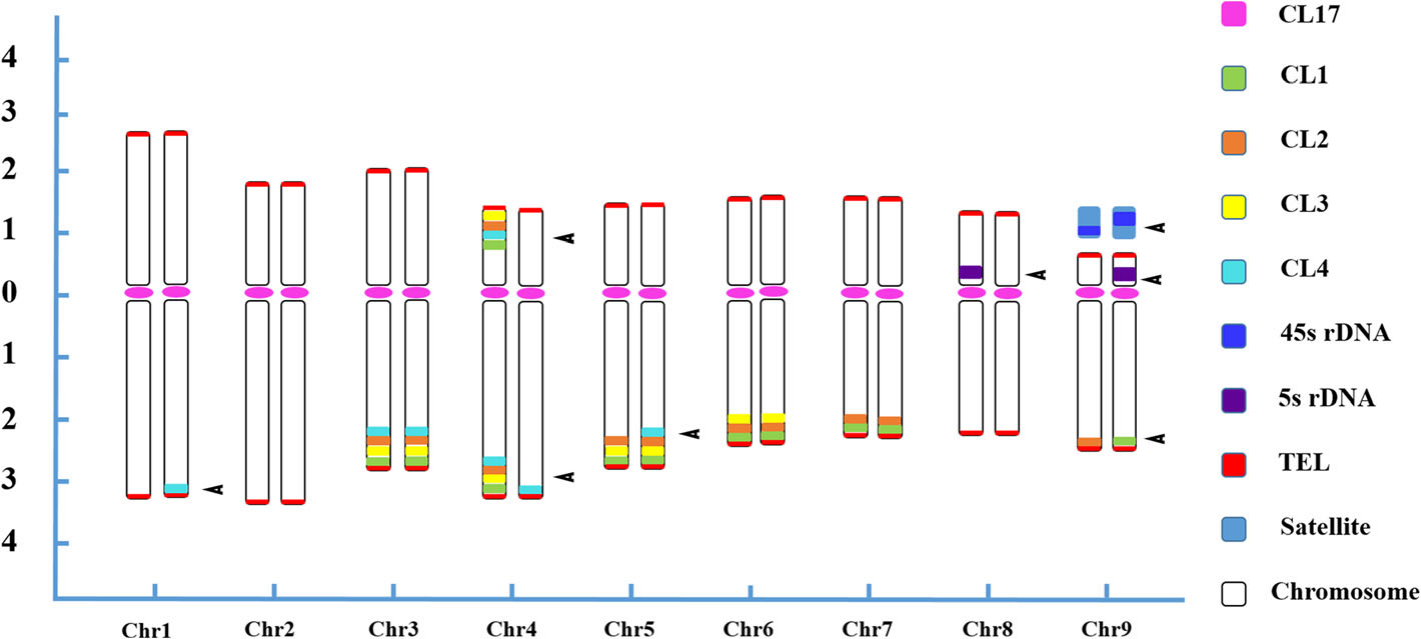
Ideograms of *C. clementina* somatic metaphase chromosomes. The short arm is positioned on top by convention. The numbers in the x-axis range from 1 to 9, indicating the correspondence of each chromosome to its homolog in Clementine. The numbers in the y-axis represent the relative chromosome length, and are based on the mean morphometric parameters from Additional file 4. Each arrowhead indicates an asymmetric pattern of the probes on homologous chromosomes

The FISH probes used in this study provided good chromosomal markers for karyotyping analysis. All individual metaphase chromosomes in Clementine were unambiguously discriminated after three consecutive rounds of multicolor FISH on the same metaphase plate (Fig. 1m). Based on the successful identification of individual chromosomes, a FISH-based integrated ideogram of Clementine metaphase chromosomes is shown in Fig. 2, in which the chromosomes were ordered and designated by descending total length at metaphase. The measurements and chromosome classification are summarized in Additional file 4. Interestingly, we detected several conspicuous asymmetrical FISH signals on the Clementine chromosomes. As shown in Fig. 2, chromosome pairs 8 and 9 carried different 5 s and 45 s rDNA signals. The characteristic features of chromosome pair 9 were a secondary constriction and a satellite that was located terminally on the short arm. A strong 45 s rDNA signal was observed in a large region of one homolog of chromosome pair 9, from the distal end of the short chromosome arm across the secondary constriction almost to the end of chromosome satellite, while another moderate 45 s rDNA hybridization signal occurred between the proximal part of the short arm and the secondary constriction; thus, 45 s rDNA produced clear signals of varying intensities and positions. Notably, chromosome pair 4 did not produce identical signals for any of the four satellite DNAs. An asymmetric CL4 locus was also observed on chromosome pairs 1 and 5, where it produced only one FISH signal at the terminal position of the long arm of one homolog. Similarly, CL1 and CL2 generated only one hybridization signal at each end of the long arms of chromosome pair 9.

### FISH patterns of these probes in several genotypes within *Citrus* and related genera

To test the potential application of these probes to other genotypes in *Citrus* and related genera, we performed successive multicolor FISH experiments to several *Citrus* genotypes and related genera in the study. The FISH signal numbers of these probes in the tested *Citrus* species and related genera are presented in Table 2, and all detailed images are presented in Additional file 5.

**Table 2:** FISH site numbers of the repetitive DNAs in 23 investigated genotypes in *Citrus* and related genera

No.	Common name	Scientific name	CL1	CL2	CL3	CL4	45 s	5 s
1	Clementine mandarin	*C. clementina* Hort. ex Tan.	11	11	8	7	2	2
2	Honghe papeda	*C. hongheensis* Y.L.D.L.	18	18	18	18	2	2
3	Ichang papeda No.4	*C. ichangensis* Swing.	20	19	16	21	6	2
4	Ichang papeda No.2586	*C. ichangensis* Swing.	15	17	12	19	6	2
5	Mauritius papeda	*C. hystrix* D.C.	14	15	12	12	4	4
6	Ziyangxiangcheng	*C. junos* Sied. ex Tan.	13	16	10	15	4	2
7	Zhencheng	*C. junos Sied.* ex Tan.	12	12	13	13	4	2
8	Muli citron	*C. medica* L.	12	12	12	15	2	2
9	Eureka lemon	*C. limon* (L.) Burm. f.	9	9	9	9	3	2
10	Longfeng big lemon	*C. limon* (L.) Burm. f.	14	14	14	14	3	2
11	Xiangyuan	*C. wilsonii* Tan.	9	14	11	9	4	2
12	sour orange	*C. aurantium* L.	12	12	12	12	4	2
13	Changshoushatian pummelo	*C. grandis* (L.) Osbeck	16	16	15	15	4	2
14	Guanxiang pummelo	*C. grandis* (L.) Osbeck	14	14	15	16	4	2
15	Thompson grapefruit	*C. paradisi* Macf.	14	14	14	18	4	2
16	Daoxian wild mandarin	*C. daoxianensis* S. W. He	9	9	8	6	2	2
17	Tachibana	*C. tachibana* Tan.	8	10	12	7	4	2
18	Mangshan wild mandarin	*C. mangshanensis* S. W. He	10	8	10	14	6	2
19	Thin-skin trifoliate orange	*P. trifoliata* (L.) Raf.	11	10	10	10	6	6
20	Crinkle-skin trifoliate orange	*P. trifoliata* (L.) Raf.	10	10	10	8	6	6
21	Changshou kumquat	*F. obovata* Tan.	14	14	12	14	4	2
22	Citrange	*C. sinensis* × *P. trifoliata* (L.) hybrid	10	12	11	7	4	3
23	Swingle citrumelo	*P. trifoliata* (L.) Raf. × *C. paradisi* Macf.	13	14	14	13	4	4

The simultaneous localization of these probes revealed some similar distribution patterns in *Citrus*, *Poncirus*, and *Fortunella* and some variability within species even in same genus. An uneven number of FISH signals was commonly observed as shown in Table 2. The telomere DNAs are probably common to all of the tested biotypes and were located terminally on the chromosomes with 36 total FISH signals (Additional file 5). The FISH hybridization signal numbers of these probes ranged from 2 to 21, and the size, intensities and localization also showed a high degree of variation even within the same genus. In general, 230, 240, 221, and 240 signals of CL1, CL2, CL3 and CL4 loci, respectively, were detected in the 18 biotypes of *Citrus*, and each biotype had 6 to 21 of these repetitive DNA loci. There were 68 and 38 signals of 45 s rDNA and 5 s rDNA loci, respectively, in the 18 tested biotypes, including 2, 3, 4, or 6 sites in one biotype. *C. ichangensis*, *C. hongheensis* Y. L. D. L. and *C. grandis* (L.) Osbeck had the largest number of repetitive DNA loci*,* whereas *C. daoxianensis* S. W. He, *C. limon* and *C. tachibana* Tan. had the lowest number among the tested genotypes in *Citrus*. The remaining 12 genotypes within *Citrus* had numbers between these six species. There were 21, 20, 20, and 18 signals of CL1, CL2, CL3 and CL4, respectively, in the two biotypes of *Poncirus*. Both *Poncirus* biotypes had 6 45 s and 6 5 s rDNA sites. Changshou kumquat had 14, 14, 12 and 14 signals for the four satellite DNAs, respectively, with 4 45 s rDNA and 2 5 s rDNA sites. There were 23, 26, 25, and 20 signals for CL1, CL2, CL3, and CL4 in the two interspecies hybrids, citrange and Swingle citrumelo. These two hybrids both had 4 45 s rDNA sites. Citrange and Swingle citrumelo had 3 and 4 5 s rDNA sites, respectively (Table 2).

## Discussion

### Repetitive DNA-based FISH enables reliable identification of individual *C. clementina* chromosomes

Chromosomes have long been regarded as the paramount unit of heredity carrying the genetic material of multicellular eukaryotes [13]. Unequivocal and reproducible identification of individual chromosomes can lay a foundation for further cytological research as well as subsequent genomic and genetic studies [15, 16]. All *Citrus* species have relatively small chromosomes (between 2 and 4 um) with similar morphology [1]; as a result, differentiating chromosomes based on morphology is rather challenging [1, 27, 28].

A preliminary investigation on *Citrus* karyology was conducted by Krug [1] using an optical microscope. Later, the C-banding technique revealed that prominent heterochromatic blocks in *Citrus* and related genera were distinguishable in metaphase chromosomes [28]. Subsequently, *Citrus*, *Poncirus* and *Fortunella* chromosomes were divided into eight different chromosomal types according to their heterochromatic CMA^+^/DAPE^−^ fluorescence band variations in terms of both the numbers and distribution [26, 27, 29–32]. The combination of CMA banding and rDNA-based FISH was broadly explored to provide additional chromosome landmarks [22, 23, 25–27, 29, 33]. These previous cytogenetic studies detected the existence of chromosome heteromorphism in *Citrus* and related genera, and also identified several clusters of chromosome pairs. Nevertheless, they were unable to identify and distinguish all individual chromosome pairs [26, 31]. In this study, we reanalyzed the repetitive DNAs of Clementine. Several repetitive DNAs used as FISH probes in our study were effective cytogenetic markers that enabled unambiguous identification of individual chromosomes in Clementine. Thus, a detailed karyotype of Clementine was established using a combination of chromosome measurements and repetitive DNA element-based FISH signals (Figs. 1, 2). We are now able to present the first detailed karyotype for Clementine and demonstrate its molecular cytogenetic characterization.

### Distribution characteristics of repetitive DNAs in *Citrus* and related genera

Many studies have shown that repetitive DNAs are functionally important for eukaryotic genomes through amplification, deletion and differentiation [9]. Satellite DNAs comprising head-to-tail tandem repeats are believed to be the most dynamic components [17, 18], undergoing the most rapid changes in the number and position of sites within a short evolutionary period [8, 10, 34]. Here, the newly developed software package RepeatExplorer was employed for the bioinformatics analysis of repetitive elements in Clementine, as it has been extensively used to characterize repetitive elements in several plants [17, 18, 35, 36].

The failure of RepeatMasker to identify satellite repeats prompted us to reanalyze the repetitive elements in the Clementine genome [3]. Our bioinformatics analysis showed that repetitive DNA elements constituted as much as 41.95% of the whole Clementine genome. Similar results (45%) were previously published for the Clementine genome, with different LTR-retrotransposon elements contents and numerous uncharacterized elements [3]. One possibility might be that satellite DNA is the most challenging part of the genome to assemble [12]. Thus, satellite elements would probably be missed or classified as other repeat elements by RepeatMasker. Here, we found that satellite DNAs are quite abundant (9.28%) in the Clementine genome. By comparison, a relatively high percentage of satellite DNAs was found in radish (*Raphanus sativus* L.) with 12.93% [18] and *Tripsacum dactyloides* with 14.66% [20], and a low percentage was found in *Coix lacryma-jobi* L. cultivar BJ with 0.60%, *Coix aquatica* Roxb. cultivar HG with 4.89% [17], and sunflower (*Helianthus annuus* L.) with 1.53% [37].

The distribution of repetitive DNAs may be related to karyotype characteristics and chromosomal organization. Here, CL17 was preferentially located in centromeric regions in Clementine (Fig. 1c), which dramatically increased the accuracy of determining the centromere positions of individual chromosomes and helped to characterize the features of homologous chromosomes. The telomere-associated repeat monomer 5′-TTTAGGG-3′ had a nonrandom distribution in the terminal region and tips of Clementine chromosomes (Fig. 1k), indicating that the nine homologous Clementine chromosome pairs shared the same telomere-associated repeat monomer [38]. Further support for this conclusion came from similar patterns of telomere repeats in several other plant species [17, 18, 38]. The 5′-TTTAGGG-3′ represents the basic and canonical telomere sequence of higher plants in a chromosome-specific manner [38]. These two repetitive DNAs are located at the centromeres and telomeres of Clementine, respectively, which potentially reflects their vital structural and functional roles in chromosome protection and nuclear organization [10, 26].

We found that 45 s rDNA sites were commonly located in the terminal regions of the short arms carrying a secondary constriction in Clementine (Figs. 1, 2). In many cases, 45 s rDNA was also observed to be restricted to nucleolus organizer regions and the telomeric regions of short arms in several other genotypes investigated here (Additional file 5). An analogous situation has also been reported in several previous studies in other *Citrus* species, as the activity of the 45 s rDNA genes is usually associated with nucleolus organizing regions and secondary constrictions [33, 39–41]. Adjacent 45 s and 5 s rDNA sites at the terminal position of the same chromosome short arm were found in the karyotype of *C. clementina* (Figs. 1, 2) and many other species investigated in this study (Additional file 5), indicating that 45 s and 5 s rDNA loci might be positively correlated and reflect the preferential distribution of the two sites in this arm [42]. The FISH patterns of 45 s rDNA loci were more polymorphic than those of 5 s rDNA loci, as almost all investigated species had two 5 s rDNA loci except Mauritius papeda (*C. hystrix* D. C.), which had four loci (Table 2).

At the molecular level, satellite DNAs are the predominant components of heterochromatin, and are typically associated with centromeric, pericentromeric, subtelomeric and telomeric regions of chromosomes [9, 10, 18, 19, 34, 43]. The cytogenetic mapping results of the present study showed that four satellite DNAs were preferentially distributed in the subtelomeric and certain telomeric distal regions of Clementine chromosomes (Fig. 1b, d, f, h). They showed different site numbers in Clementine and other genotypes; however, favoring the terminal location can be regarded as a general tendency of their chromosomal distributions (Figs. 1, 2, Additional file 5), which is within the range of the extensively reported distribution patterns. In general, the satellite DNA sites showed much more variability both in numbers and physical chromosomal localization than did the rDNA sites in this study (Table 2). Although the reason for these preferential distributions of repetitive DNAs is not entirely known, several possible explanations have been postulated in the literature. For example, the existence of satellite DNAs without a preferential centromeric or pericentromeric distribution in the tested genotypes in *Citrus* and related genera possibly indicates that multiple sorts of retrotransposons comprise centromeric regions [19]. Another potential explanation is that the terminal-preference distribution is related to the regulation of chromosome stabilization or disjunction in mitosis and meiosis [8, 10]. Furthermore, the observed trends may be related to a dynamic process [14, 31]. According to Garrido-Ramos [10], plant satellite DNAs are commonly structured as heterochromatin, which frequently exists in pericentromeric and subtelomeric regions.

The partially or entirely overlapping signals of the probes detected in this study are consistent with reported cases of linked 5 s–45 s rDNA sites [33], co-localization of the CMA^+^ band and 45 s rDNA sites [27, 31, 39] or satellite DNAs [26] in several biotypes of the subfamily Aurantioideae (Rutaceae). In previous studies, the possible reason for the linkage of these sites was assumed to be that this is the ancestral condition [33], while unlinked sites might reflect that these repetitive DNA elements remain mobile during species evolution [27, 33].

### Implication of repetitive DNA-based FISH for the hybrid identification of *Citrus* and related genera

Despite the wide variety of available tools, hybrid identification still continues to be challenging in *Citrus* [4]. To explore the utility of repetitive DNA-based FISH among hybrid identification, we applied these repetitive DNA probes to some presumed hybrids in *Citrus* species and interspecific hybrids, including citrange and Swingle citrumelo. The molecular cytogenetic method of repetitive DNA-based multicolor FISH described here enabled certain characteristic chromosomes to be identified in species and hybrids in *Citrus*, *Poncirus*, and *Fortunella* (Table 2, Additional file 5).

In theory, homologous chromosomes should have identical FISH distribution patterns, but here the FISH signals did not always exhibit parallel patterns in both homologous chromosomes. Conspicuously, the FISH assays of 23 investigated samples in the genus *Citrus* and related genera demonstrated that uneven and nonhomologous signals were commonly (Table 2, Additional file 5). This is consistent with a previous view that most citrus crops are generally characterized by highly heterozygous traits [4]. The cytogenetic data also confirmed the widely accepted belief that many or even most *Citrus* species are derived from natural (spontaneous) or man-made (artificial) hybridization [3]. For example, the asymmetrical FISH signals with an odd number of loci could certainly provide ample evidence that the Clementine is of hybrid origin, consistent with previous studies (Figs. 1, 2, Table 2). Clementine is considered to have most likely originated from a spontaneous hybrid from China and been selected by father ‘Clement’ from Algeria over a century ago [6, 44]. On the basis of morphological characteristics, Swingle has demonstrated that the Clementine is a cross between *C. deliciosa* Ten. and *C. aurantium* L. [45]. A later serological study showed that Clementine is most closely related to ‘Baladi’ mandarin and ‘Baladi’ blood orange [46]. Molecular markers support the assumption that Clementine is a hybrid between mandarin and sweet orange [47]. The hypothesis of a Mediterranean mandarin × sweet orange was confirmed via single nucleotide polymorphisms (SNPs) despite one locus out of 506 suggesting incompatible genotypes [48]. Wu et al. [3] investigated the conjecture at the sequence level by definitively identifying a willowleaf mandarin (*C. deliciosa*) and sweet orange (*C. sinensis*) allele at each Clementine locus. The type of cytological information obtained in our present work would be of great assistance in investigating the hybrid origin of Clementine. However, we did not confirm or discuss its putative parents. This work will be a topic of future research. However, our study allows us to conclude that heteromorphism may be an apparent indication of hybridism in Clementine. Importantly, the karyotyping analysis in our study could provide direct visual proof of the heterozygous nature of Clementine chromosomes.

The similar repetitive DNA distribution patterns in other species and hybrids within *Citrus*, *Poncirus* and *Fortunella* in this study are consistent with the conclusion that notable conservation exists between the three genera [49]. An intriguing interpretation is that the three closely related genera originated from the genome of a common ancestor before speciation, which has been conserved in the genomes of *Citrus* plants because of a possible role of satellite DNAs in heterochromatin organization [22, 40]. Additionally, polymorphism in site numbers and variation in chromosomal locations of repetitive DNA loci is commonly observed in the three genera (Table 2). Similar results have been reported showing that *Citrus* chromosomes exhibit a high degree of diversity and heterozygosity, through which we can obtain further essential information to shed light upon the phylogenic and taxonomic relationships of *Citrus* and related genera [26, 27, 29, 33]. A plausible explanation for the observed chromosome distribution variations is the rapid amplification and/or reduction of repetitive elements [9, 34].

From the perspective of molecular cytogenetics, we found that biotypes with more signals were more ancestral, while those with fewer signals were more evolved or of hybrid origin. For example, *C. hongheensis* from the subgenus *Papeda* (Swing.) has been paid much attention in both the Swingle and Tanaka systems [2, 45]. Here, *C. hongheensis* showed the same number of CL1, CL2, CL3, and CL4 sites, unlike the other species. The ancient species *C. mangshanensis* S. W. He displayed more FISH signals than the other mandarin genotypes investigated. As mentioned above, 45 s rDNA FISH loci showed numerical variation from two to six sites, whereas 5S rDNA presented a conserved number of sites (two) in *Citrus* except *C. hystrix* (Table 2). The great similarity of *C. hystrix* was demonstrated by Pang et al. [50] with a relatively large content of polymorphic AFLP fragments and by Zhou et al. [51] with clustering data on its morphological characteristics, which co-contributed to the identification of its hybrid origin. The molecular cytogenetic data presented here might serve as a starting point for further elucidation of the karyotype evolution and the taxonomical relationships of *Citrus*, *Poncirus*, and *Fortunella* at a larger scale in the future.

## Conclusions

To summarize, the composition and abundance of repetitive elements in the Clementine mandarin (*C. clementina*) genome were reanalyzed. A set of several probes that contained CL17, four major satellite DNAs, two rDNAs, and the telomeric repeat TTTAGGG was used to develop a molecular cytogenetic karyotype of Clementine somatic metaphase chromosomes. Unequivocal identification of individual Clementine chromosomes was achieved. Multicolor FISH-based karyotyping provided direct visual proof of the heterozygous nature of Clementine chromosomes, which showed conspicuous asymmetrical FISH hybridization signals. In addition, we detected some similar and variable distribution patterns in 23 genotypes within *Citrus*, *Poncirus*, and *Fortunella*, which revealed notable conservation among these genera, but also obvious polymorphism and heterozygosity, providing an indication of the potential utility of these repetitive element markers for the study of the taxonomic, phylogenetic and evolutionary relationships in the future.

## Methods

### Plant materials and genomic DNA extraction

The materials (Additional file 6) were provided by the National Citrus Germplasm Repository in Chongqing, China. Root tips were harvested from germinated seeds obtained from open-pollinated mother plants. To increase the chance of working with nucellar rather than zygotic embryos, five root tips from seedlings of each species were preferentially analyzed individually, and only those that exhibited an identical karyotype in at least three root tips were accepted for analysis as nucellar embryos [23, 29]. If the karyotype was repeated in at least three seedlings, it probably represented the nucellar and maternal karyotype. Clementine genomic DNA was extracted by the CTAB protocol using young leaves. The genome information for Clementine was publicly available from the website https://www.citrusgenomedb.org/species/clementina.

### Repeat composition analysis of *C. clementina*

RepeatExplorer, which has a graph-based sequence-clustering algorithm, was used to identify repetitive elements de novo and explore their proportional composition in the Clementine genome in more detail [36]. The clustering analysis was performed using a cluster size threshold of 0.005% [35, 36]. Sequencing data were first preprocessed to remove low-quality reads using ‘filter by quality’ (available in RepeatExplorer), and then the unpaired reads were dropped using FASTO interlacer (also available in Repeat Explorer). From the filtered data, 99,500 reads (100,495,000 bp) were randomly selected. Repeats were identified de novo using a similarity-based reads clustering method. Reads within individual clusters were also assembled into contigs representing sequence variants of corresponding repeats. Basic repeat classification was performed using a combined approach including the examination of cluster graph shape, similarity searches of DNA and protein databases, and the detection of subrepeats in assembled contigs. Typically, clusters with satellite DNA had a star-like and circular graphical representation. The cluster graph topology and occurrence of subrepeats in contigs were also primary criteria for identifying tandem repeats. To classify the putative satellite monomers in the individual clusters, the assembled contigs were subjected to the Tandem Repeat Finder software [52].

### Chromosome preparation, probe labeling and FISH

Chromosome preparation, probe labeling and FISH procedures followed the protocols described by Cai et al. [17] with minor modifications. Roots were harvested and immediately exposed to nitrous oxide under approximately 150 PSI pressure for 2 h, fixed in Carnoy’s solution and ethanol-acetic acid (*v*/v, 3:1) for 2–24 h at room temperature, and stored at − 20 °C until required. The root tips were cut into a fine length after washing in distilled water to remove the above fixative solution and macerated in an enzyme mixture solution containing 3% (*w*/*v*) Cellulase and 0.3% (w/v) Pectolyase at 37 °C for 1.5 h. Then, the enzyme solution was removed and a drop of distilled water was added for a 10 min hypotonic treatment, after which the water was removed and fresh Carnoy’s solution was added. Finally, the samples were smashed on ice-cold slides and dried on a flame.

DNA probes for satellite repeats and rDNA were amplified by PCR from *C. clementina* genomic DNA. Cloning of the satellite repeats and rDNA was performed using primers designed from extracted repeat clusters. The plasmids were labeled by a nick translation reaction using biotin-16-dUTP, digoxigenin-11-dUTP, Diethylaminocoumarin-5-dUTP and CY5-DUTP. An oligonucleotide of (TTTAGGG)_3_ was labeled at the 5′-end with digoxigenin to detect telomere repeat locations. Chromosomes were counterstained with DAPI in Vectashield antifade solution.

The successive FISH procedure was modified from a previously published protocol with minor modifications [17]. After the first round of FISH and image capture, the slides were washed twice with Clearing Agent (SIGMA), a set of PBS (phosphate buffer saline) buffer, and an ethanol series (70, 95, and 100%), and then fixed in Carnoy’s solution, ethanol-acetic acid (v/v,3:1) and 4% (w/v) paraformaldehyde before hybridization with the set of probes.

### Karyotype analyses

Chromosome cytological measurement and karyotype analysis followed the procedure described in previous studies [17, 18, 20]. A Sensys CCD camera (QIMAGING, RETIGA-SRV, FAST 1394) attached to an Olympus BX61 epifluorescence microscope (Tokyo, Japan) was used for FISH image acquisition. The chromosomes and the hybridization status of the probes used were captured under different fluorochrome channels. Mitotic metaphase images were superimposed, pseudocolored and merged in the Image-Pro plus 6.5 software (Media Cybernetics). The automatically merged images were measured using the ImageJ software (National Institutes of Health, Wayne Rasband, MD, USA). The overall chromosome size was estimated by measuring the lengths of both the short and long arms of the chromosomes, excluding satellites and nucleolar organizer regions. Homologous chromosomes were identified on the basis of chromosome lengths, morphological features, and repetitive DNA FISH signals. Final images were adjusted, and the chromosomes were organized in decreasing order by the Adobe Photoshop CS6 software. Ideograms were drawn based on the measurements and FISH signals.

## Additional files

Additional file 1: List of the annotations and genome proportion of clusters in *C. clementina*. (XLSX 28 kb)

Additional file 2: Summary of the major satellite DNAs in the *C. clementina* genome. (DOCX 13 kb)

Additional file 3: Sequences of the primers used for PCR amplification in this study. (DOCX 13 kb)

Additional file 4: The original data of the *C. clementina* karyotype analysis. (XLSX 16 kb)

Additional file 5: FISH distribution patterns of the repetitive DNAs in 23 investigated genotypes in *Citrus* and related genera. (DOCX 4526 kb)

Additional file 6: Information on the plant materials used in this study. (XLSX 10 kb)

## Abbreviations

CCD: Charge-coupled device
CMA: Chromonycin A3
CTAB: Cetyltrimethyl ammonium bromide
DAPI: 4′6-Diamidino-2-phenylindole
ESTs: Expressed sequence tag
FISH: Fluorescence in situ hybridization
ICGC: International Citrus Genome Consortium
LINE: Long interspersed nuclear element
LTR: Long terminal repeat
PBS: Phosphate buffer saline
rDNA: Ribosomal DNA
SSR: Simple sequence repeat
