# Supplementary material for: Molecular cytogenetic analysis of genome-specific repetitive elements in Citrus clementina Hort. Ex Tan. and its taxonomic implications
Source: BMC Plant Biol. 2019 Feb 15;19:77. doi: 10.1186/s12870-019-1676-3 (PMC6377768; doi:10.1186/s12870-019-1676-3)
Supplement: Supplementary file 2 — Summary of the major satellite DNAs in the C. clementina genome. (DOCX 13 kb) [file 12870_2019_1676_MOESM2_ESM.docx]

**Additional file 2**

Summary of the major satellite DNAs in the *C. clementina* genome

| Type | Total length[bp] | Number of reads | Genome proportion [%] | Monomer repeats length [bp] |
| --- | --- | --- | --- | --- |
| CL 1 | 3186550 | 31550 | 3.16 | 185 |
| CL 2 | 3039696 | 30096 | 3.01 | 185 |
| CL 3 | 1562268 | 15468 | 1.55 | 124 |
| CL 4 | 1217656 | 12056 | 1.21 | 132 |
