# Supplementary material for: Molecular cytogenetic analysis of genome-specific repetitive elements in Citrus clementina Hort. Ex Tan. and its taxonomic implications
Source: BMC Plant Biol. 2019 Feb 15;19:77. doi: 10.1186/s12870-019-1676-3 (PMC6377768; doi:10.1186/s12870-019-1676-3)
Supplement: Supplementary file 3 — Sequences of the primers used for PCR amplification in this study. (DOCX 13 kb) [file 12870_2019_1676_MOESM3_ESM.docx]

**Additional file 3**

Sequences of the primers used for PCR amplification in this study

| Repeats name | Primer type | Primer sequence (5'-3') |
| --- | --- | --- |
| CL1 | F | CCGCAAAGTCTCGGGCCAT |
| CL1 | R | CGCCCAAAAATTAGCGCCCGAAG |
| CL2 | F | TCGGAATGGCGCGAGACTTT |
| CL2 | R | GGCCTTTTTCCGCTGGACGA |
| CL3 | F | CTGCGCGCGATGGTGCCTC |
| CL3 | R | GCGCGAAACTAGCCCGCCAAC |
| CL4 | F | TCATGCCCATTTTTCGGCGTTC |
| CL4 | R | GGGCCTCCGATTCCGTTTCC |
| 45s rDNA | F | ACTAAGAACGGCCATGCACCA |
| 45s rDNA | R | ATCCTGCCAGTAGTCATAGCTT |
| 5s rDNA | F | ACAATGTCTTCCGCCCGGATC |
| 5s rDNA | R | GGCCGAAGAGGGGAAAGGTTC |
| CL17 | F | CTGTTTGTCCATCTTCAAGGGG |
| CL17 | R | CTTTCTGTTGAGATGAGTGTCCG |
| TTTAGGG | / | (TTTAGGG)3 |

Notes:

F, forward primer

R, reverse primer
