# Supplementary material for: Molecular cytogenetic analysis of genome-specific repetitive elements in Citrus clementina Hort. Ex Tan. and its taxonomic implications
Source: BMC Plant Biol. 2019 Feb 15;19:77. doi: 10.1186/s12870-019-1676-3 (PMC6377768; doi:10.1186/s12870-019-1676-3)

**Additional file 5**

FISH distribution patterns of the repetitive DNAs in 23 investigated genotypes in *Citrus* and rela­ted genera. The chromosomes were isolated from the same metaphase cells. For a better visualization, the FISH signals in (b-d), (f-h), and (j-k) were digitally separated from the merged graphs (a), (e) and (i), respectively.

1. Honghe papeda (*C. hongheensis* Y.L.D.L.)


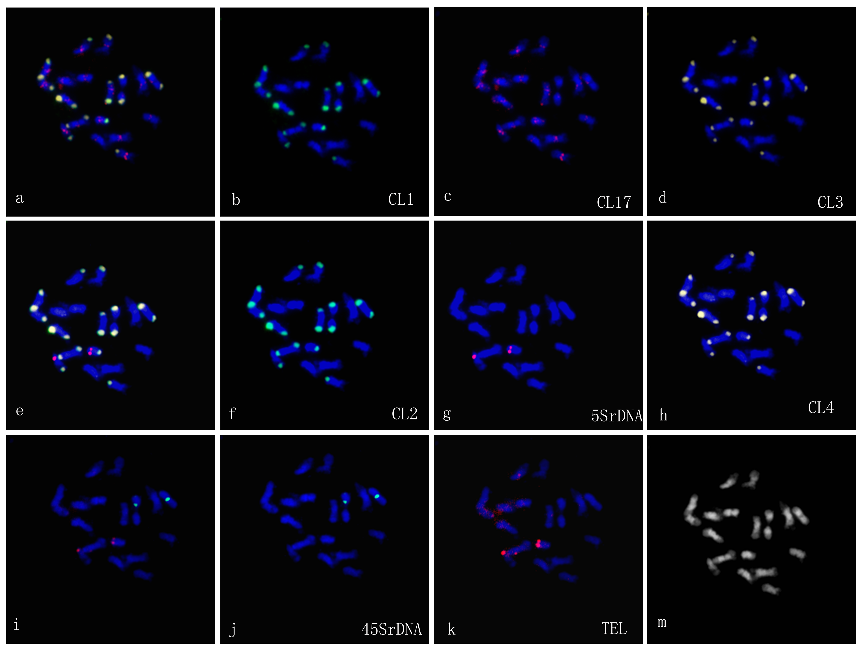


2. Ichang papeda No.4 (*C. ichangensis* Swing.)


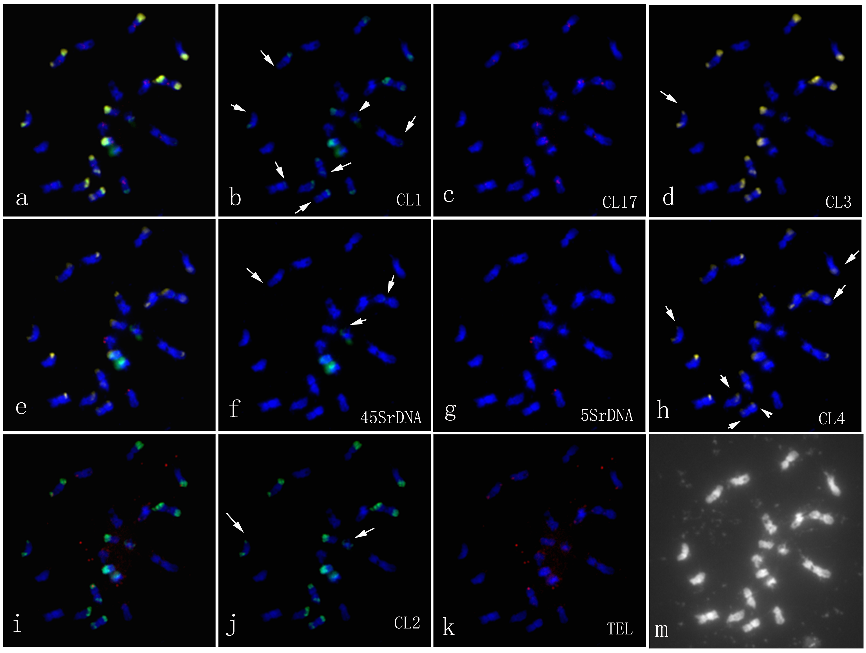


3. Ichang papeda No.2586 (*C. ichangensis* Swing.)


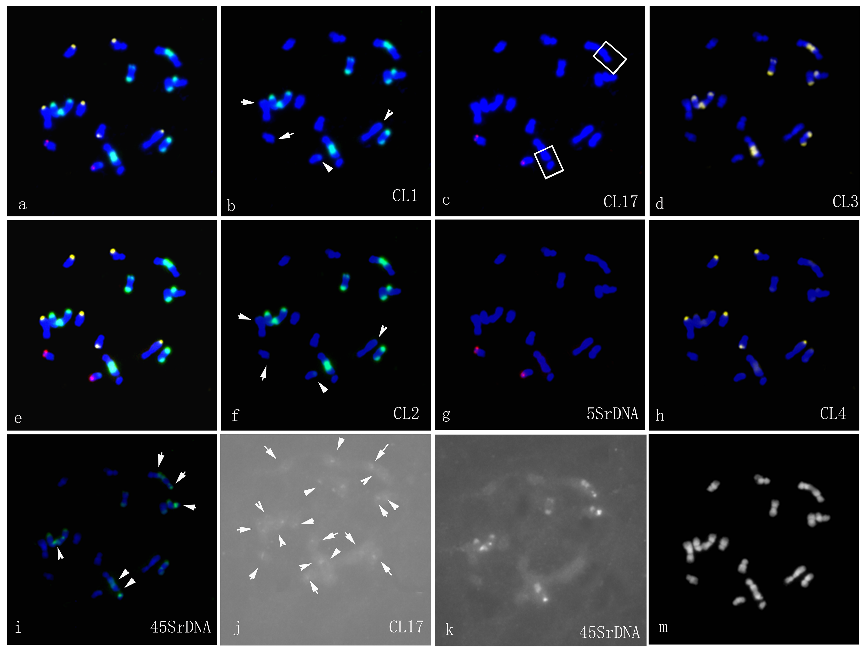


4. Mauritius papeda (*C. hystrix* D.C.)


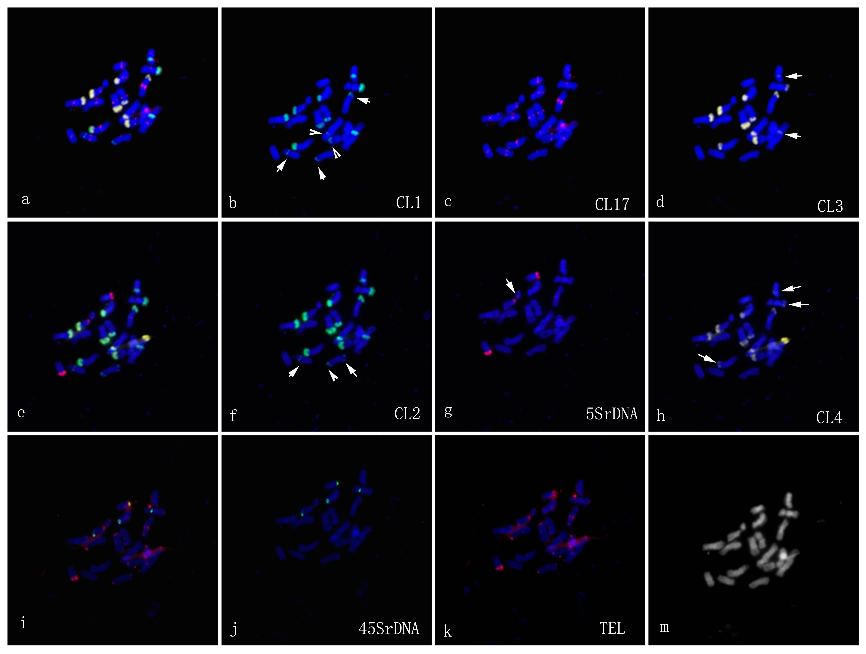


5. Ziyangxiangcheng (*C. junos* Sied. ex Tan.


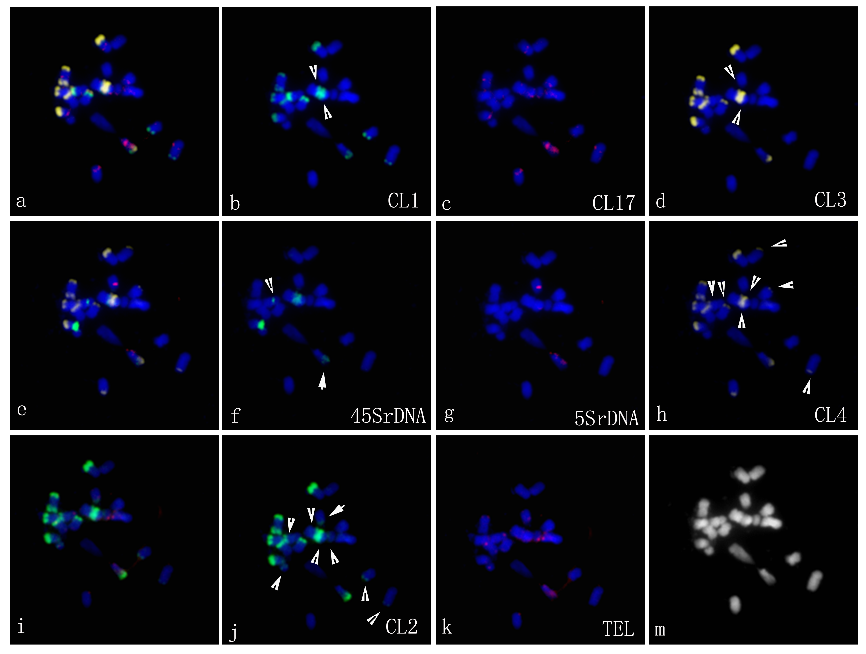


6. Zhencheng (*C. junos S*ied. ex Tan.)


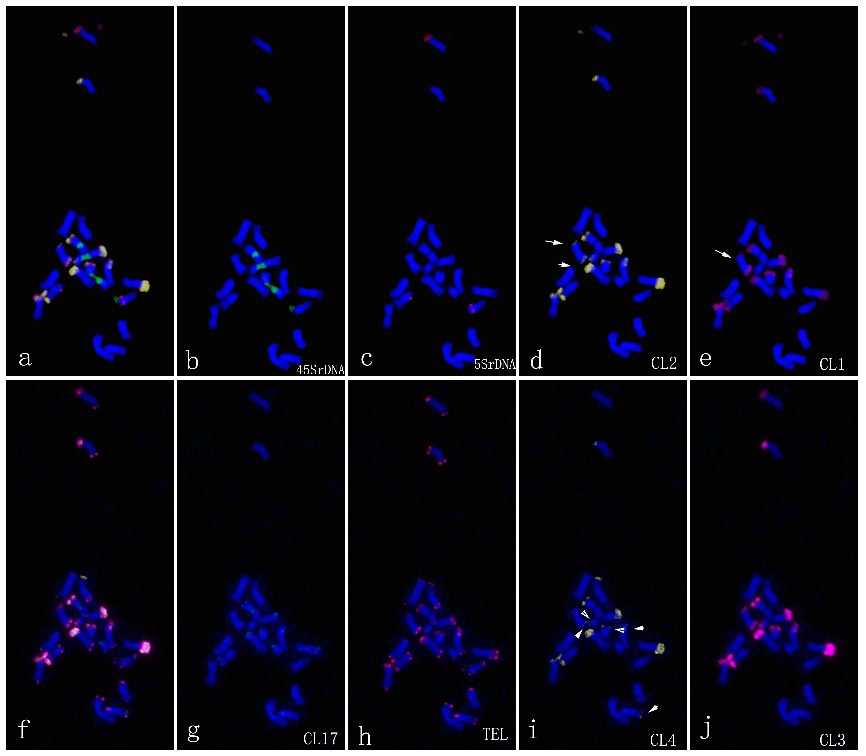


7. Muli citron (*C. medica* L.)


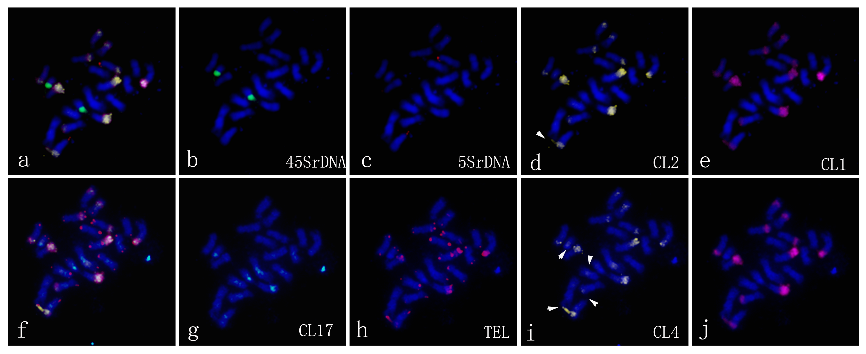


8. Eureka lemon (*C. limon* (L.) Burm. f.)


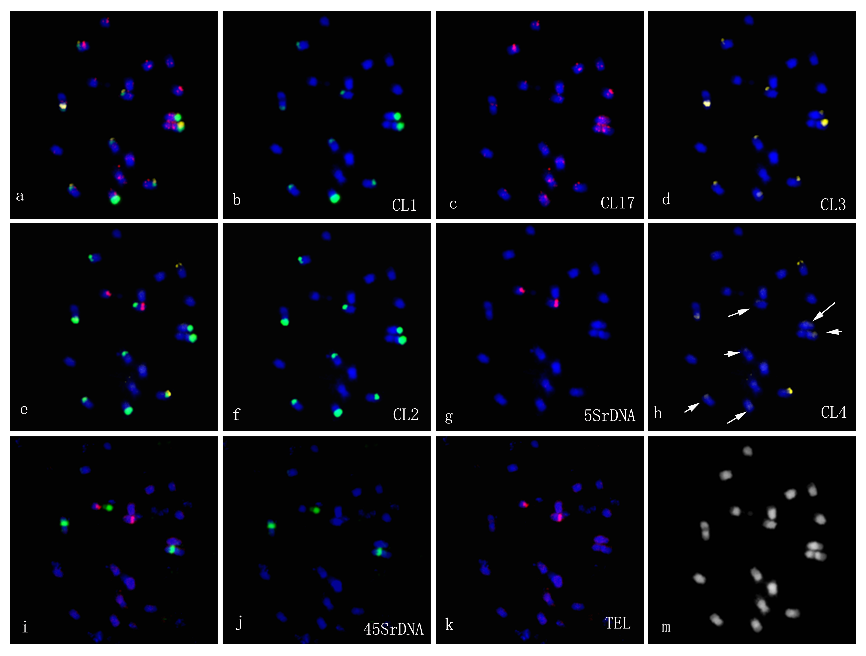


9. Longfeng big lemon (*C. limon* (L.) Burm. f.)


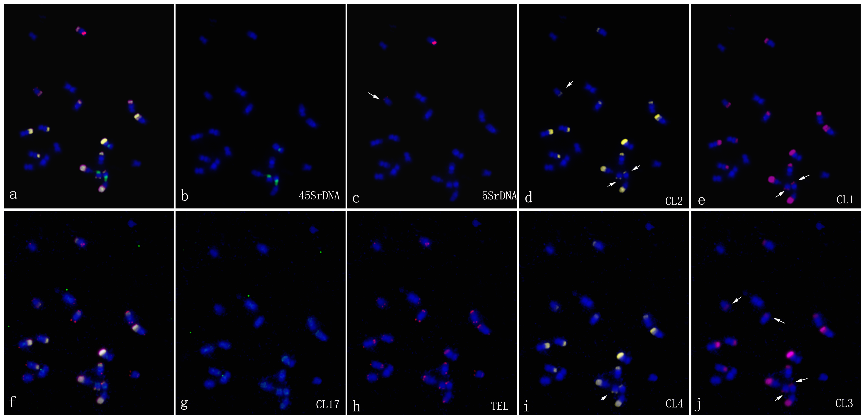


10. Xiangyuan (*C. wilsonii* Tan.)


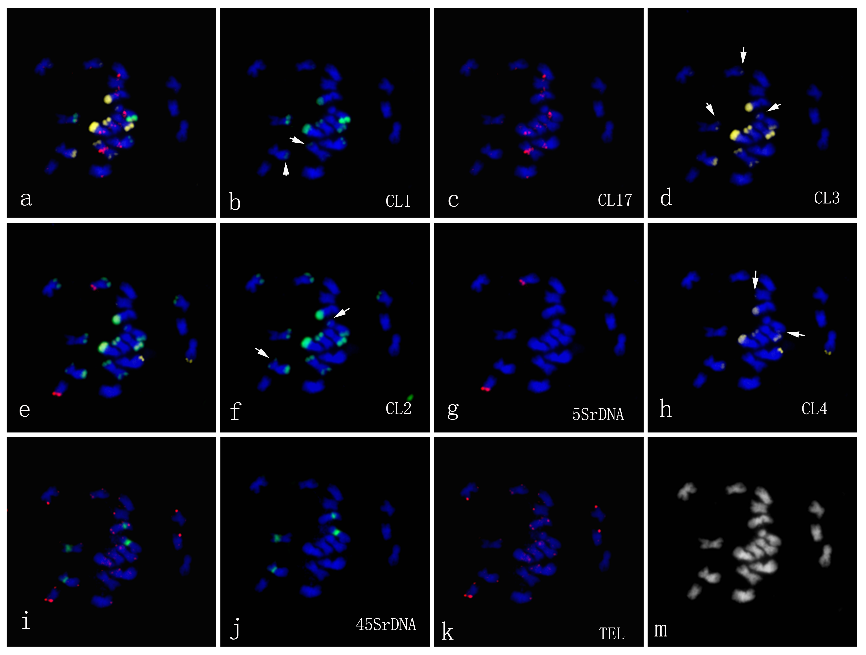


11. Sour Orange (*C. aurantium* L.)


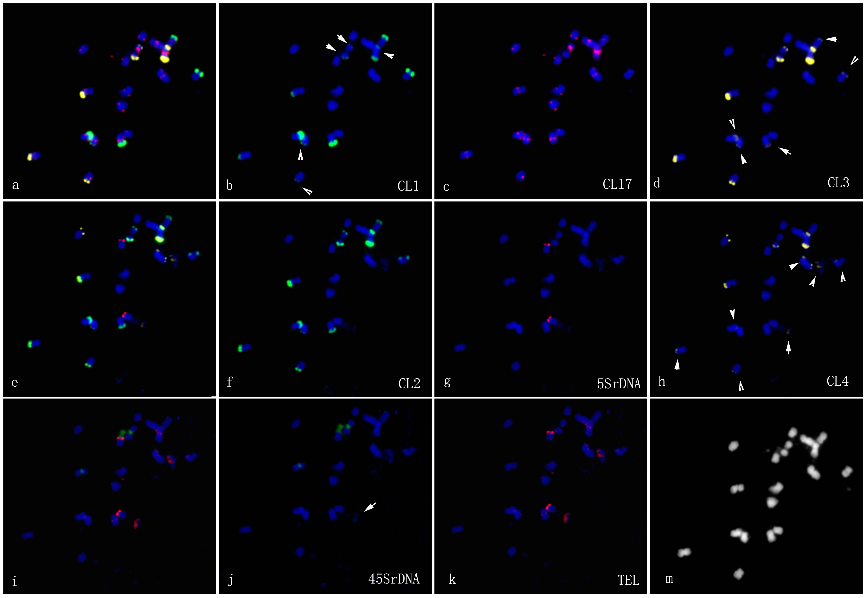


12. Changshoushatian pummelo (*C. grandis* (L.) Osbeck)


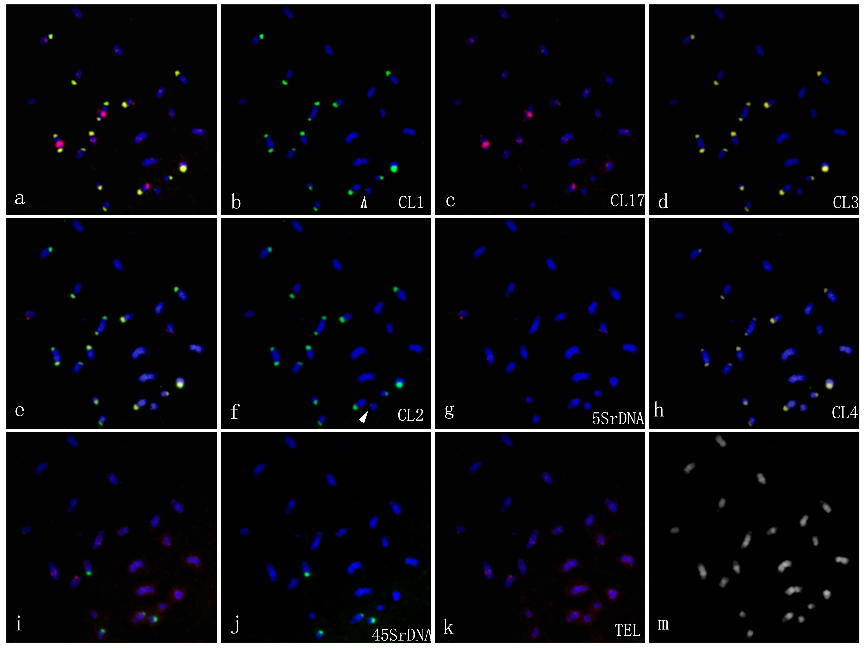


13. Guanxiang pummelo (*C. grandis* (L.) Osbeck)


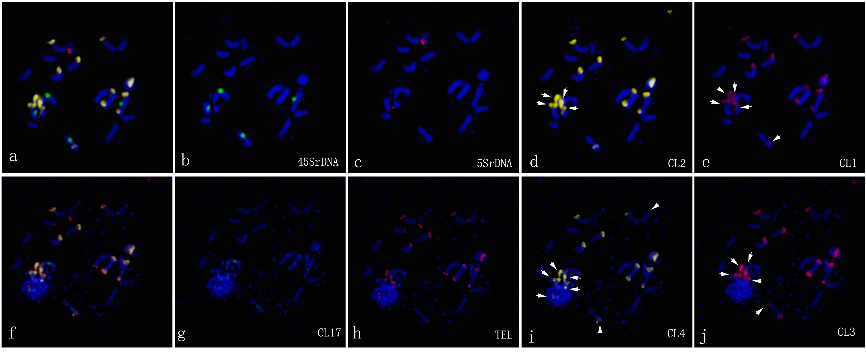


14. Thompson Grapefruit (*C. paradisi* Macf.)


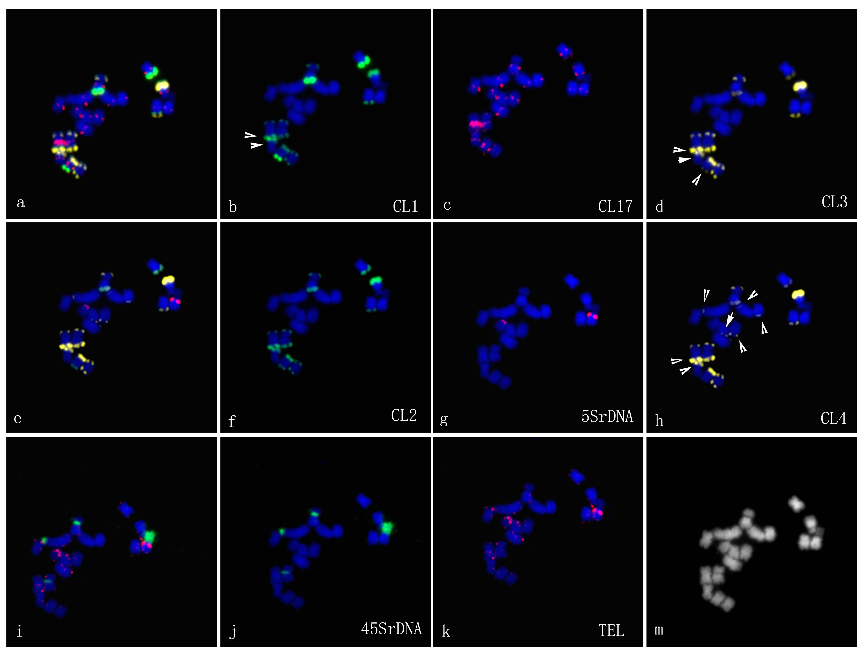


15. Daoxian wild mandarin (*C. daoxianensis* S.W.He)


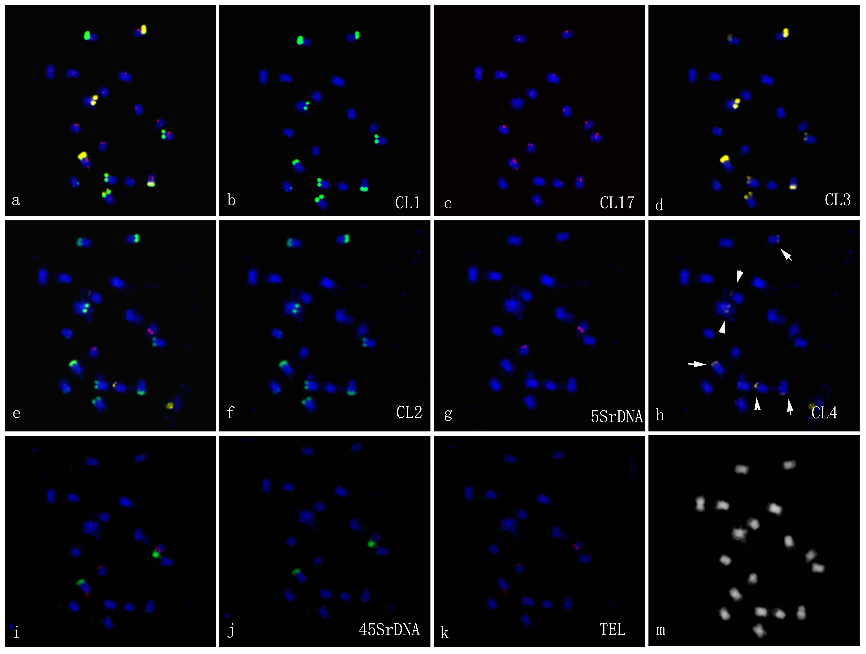


16. Tachibana (*C. tachibana* Tan.)


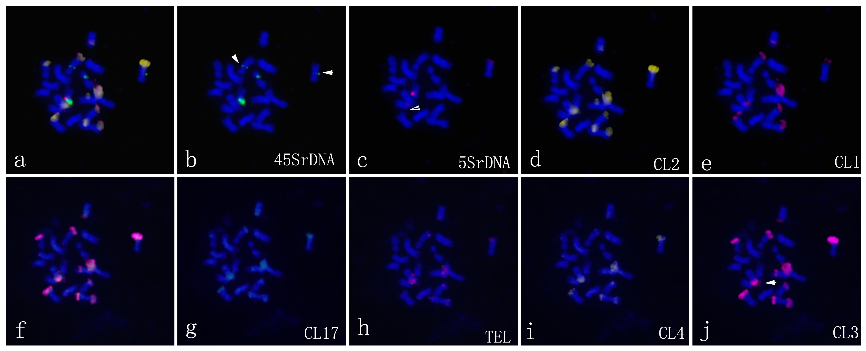


17. Mangshan wild mandarin (*C. mangshanensis* S.W.He)


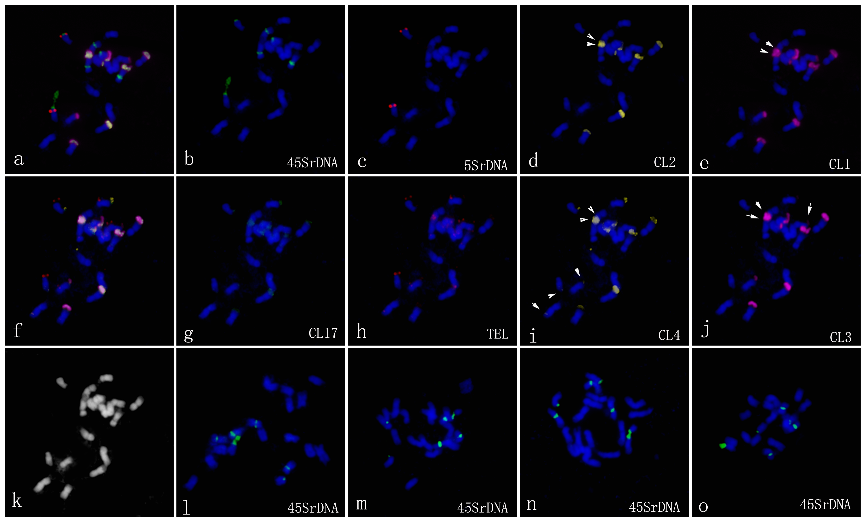


18. Thin-skin trifoliate Orange (*P. trifoliata* Raf.)


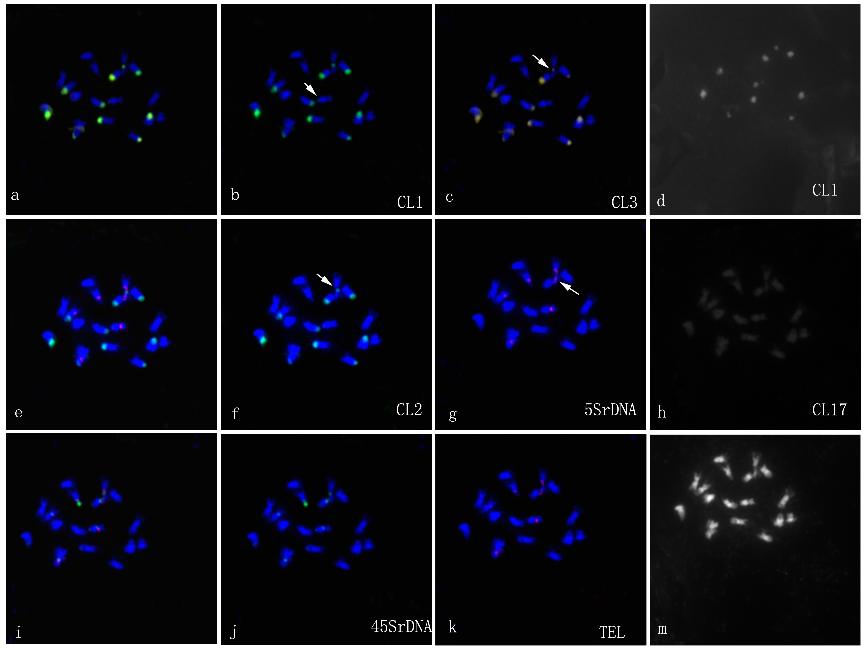


19. Crinkle-skin trifoliate orange (*P. trifoliata* Raf.)


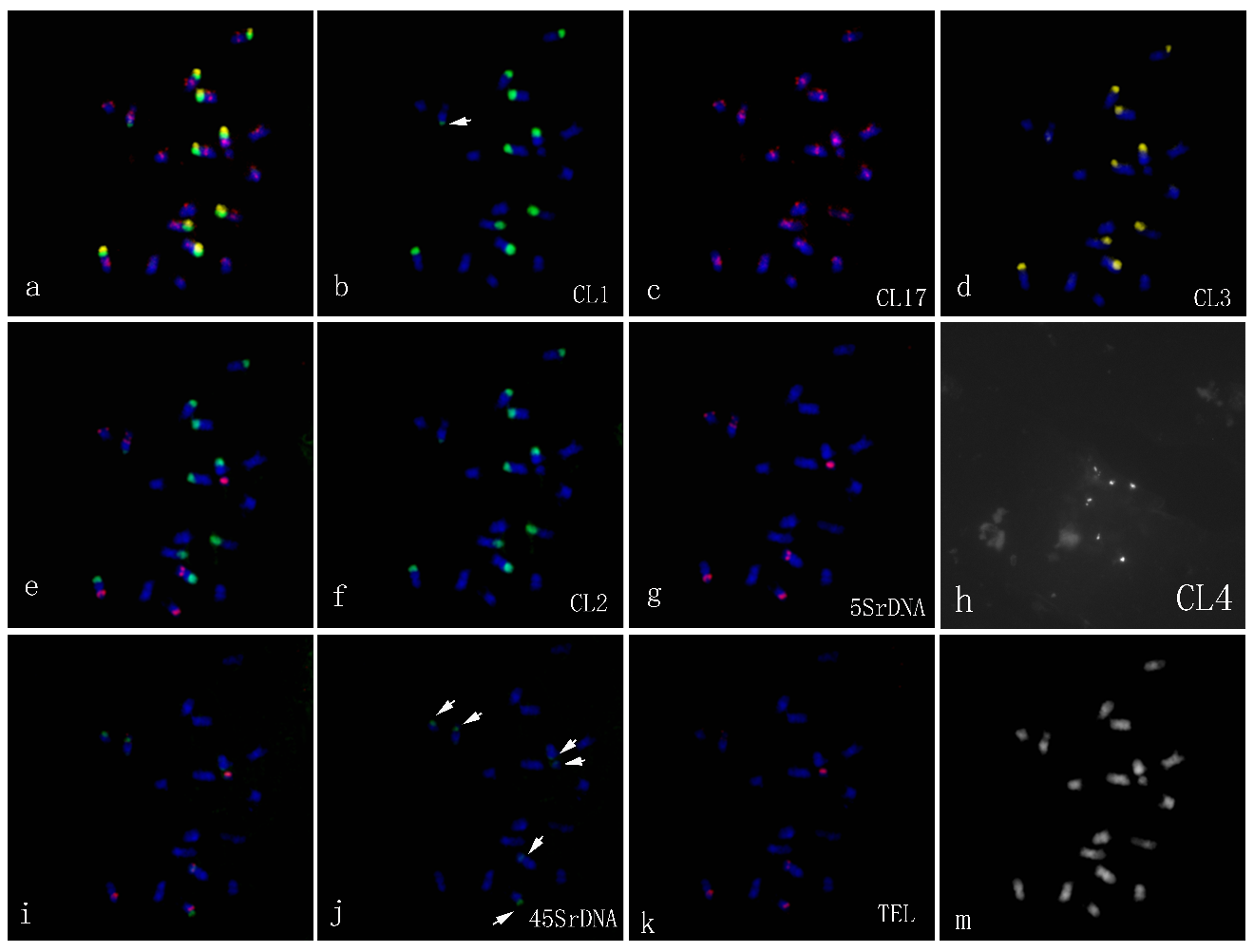


20. Changshou kumquat (*F. obovata* Tan.)


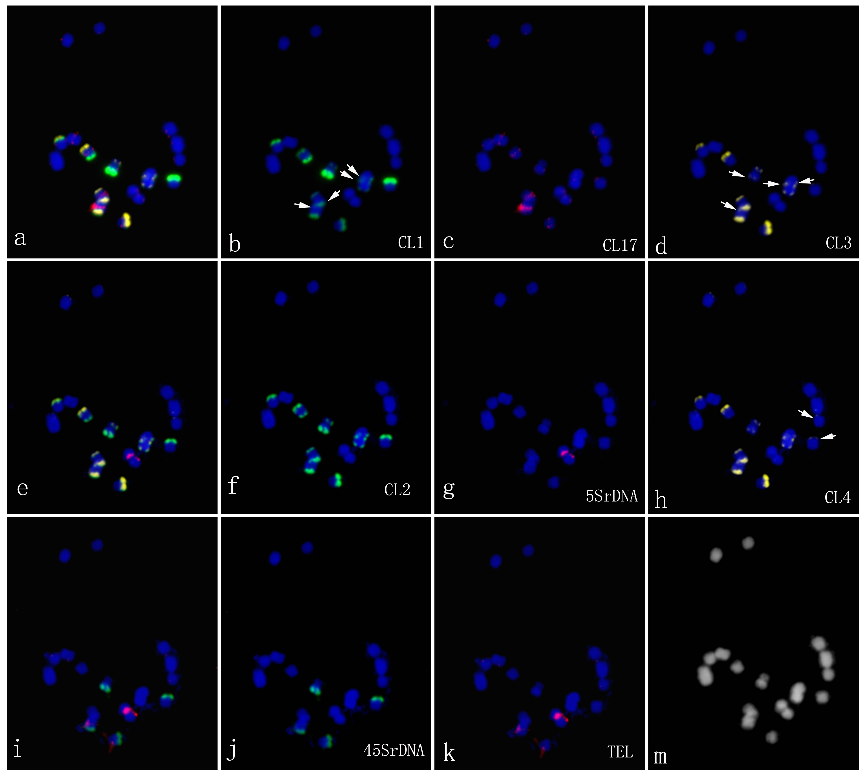


21. Citrange (*C. sinensis* × *P. trifoliata* hybrid)


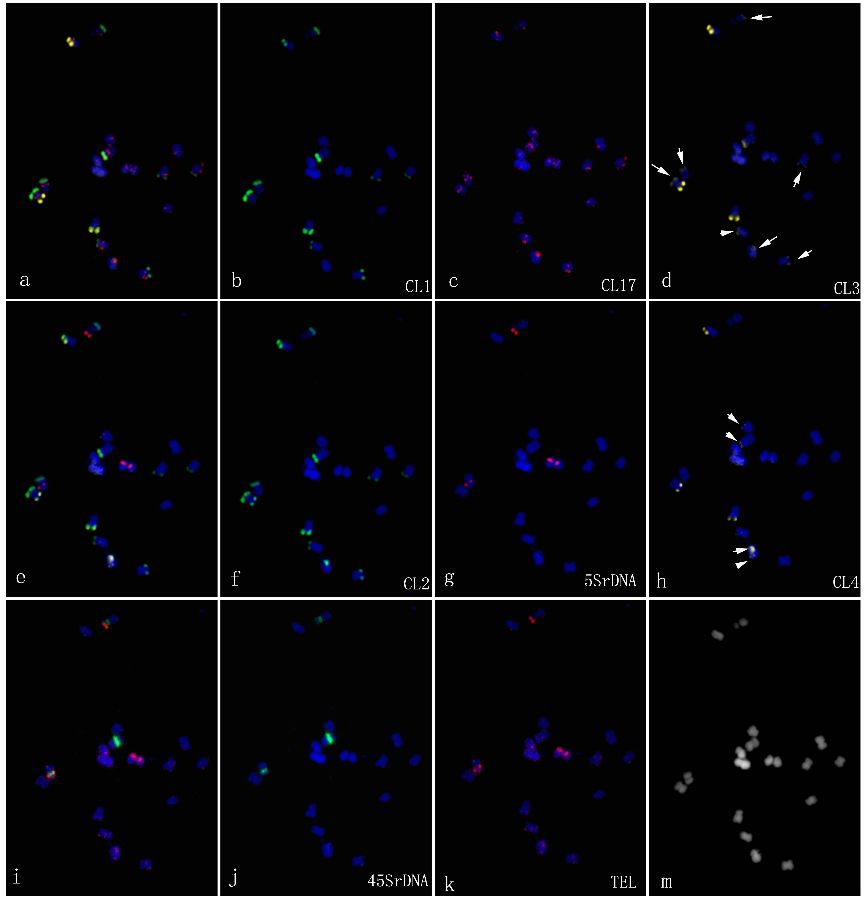


22. Swingle citromelo (*P. trifoliata* Raf. × *C. paradisi* Macf.)


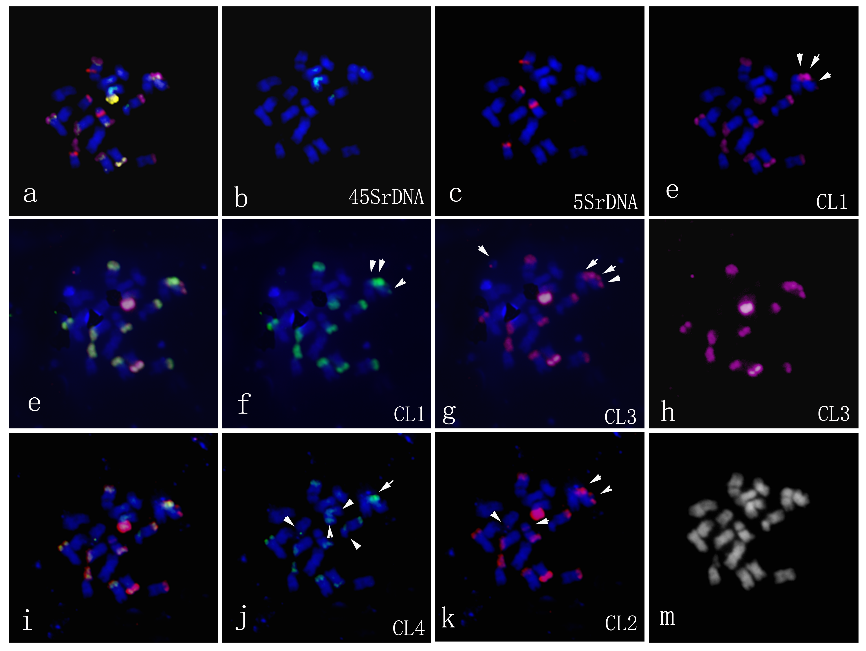

Supplement: Supplementary file 5 — FISH distribution patterns of the repetitive DNAs in 23 investigated genotypes in Citrus and related genera. (DOCX 4526 kb) [file 12870_2019_1676_MOESM5_ESM.docx]
